# MYSTAT: A compact potentiostat/galvanostat for general electrochemistry measurements

**DOI:** 10.1016/j.ohx.2020.e00163

**Published:** 2020-12-13

**Authors:** P. Irving, R. Cecil, M.Z. Yates

**Affiliations:** Department of Chemical Engineering, University of Rochester, Rochester, NY 14627, United States

**Keywords:** Electrochemistry, Cyclic voltammetry, Amperometry, Battery testing, Sensors

## Abstract

An open-source potentiostat/galvanostat instrument design is introduced that provides the ability to take accurate measurements over a current range of ±200 mA and a potential range of ±12 V. The improved capability of the instrument compared to the previously published design upon which it is based makes it suitable for performing a wider range of electrochemical measurements including the ability to use larger working electrodes, study of high current density processes, study of electrochemistry in nonaqueous solutions and use in high voltage processes such as electrophoretic deposition. The instrument can be controlled from any computer capable of running the Python programming language, including a low-cost Raspberry Pi. Unlike many commercial potentiostat designs, the instrument is completely open-source, giving researchers the ability to modify the hardware and software as needed for custom measurement techniques. The low cost makes the instrument attractive for research and teaching laboratories in which multiple electrochemical measurements need to be carried out in parallel.

## Graphical abstract

**Figure:**
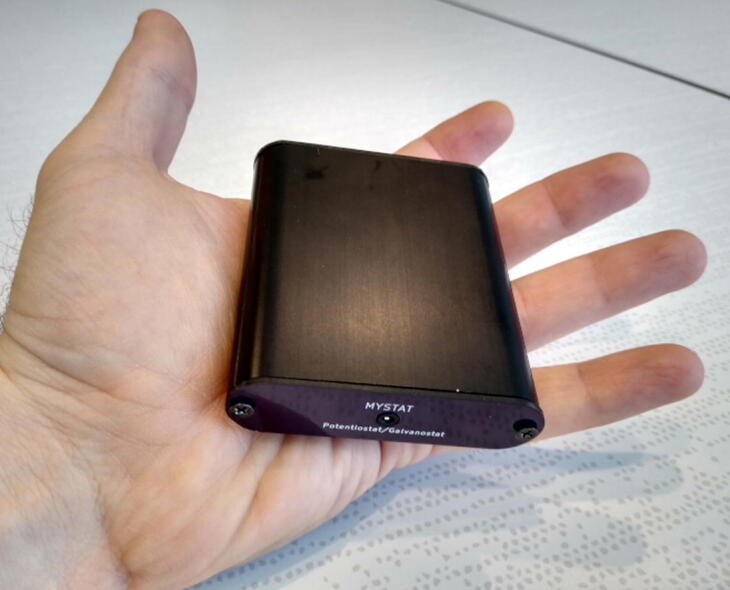

Specifications table

**Table:** 

Hardware name	MYSTAT potentiostat/galvanostat
Subject area	• Engineering and Material Science • Chemistry and Biochemistry
Hardware type	• Measuring physical properties and in-lab sensors • Electrical engineering and computer science
Open Source License	GNU General Public License v.3
Cost of Hardware	$226.22
Source File Repository	http://doi.org/10.5281/zenodo.4252476

## 1 Hardware in context

A potentiostat is a device that is used to control the potential of an electrode by adjusting the electrical current supplied. Unlike a simple direct current power supply or fixed voltage source, the potentiostat allows the potential of an electrode to be measured independently from the circuit used to supply current to the electrode. A galvanostat is a device that is used to control the electrical current supplied to an electrode by adjusting the applied potential. Galvanostats allow for control and measurement of both positive and negative currents through the electrode. Instruments that provide potentiostatic and galvanostatic control are fundamental to nearly all electrochemical measurements [1]. Applications of such instruments are wide-ranging and include the characterization of batteries, fuel cells, biosensors, corrosion, environmental monitoring, and the electrochemical synthesis of materials. Commercial electrochemical test equipment typically allows for switching between potentiostatic and galvanostatic modes of operation using a single instrument.

Potentiostats connect to two external circuits, one of which is used for potential measurement, the other of which is used for the application of electrical current. Current flows from the instrument through a circuit that has an external working electrode (WE) and a counter electrode (CE). The WE is the electrode under investigation by a measurement. Current flow through the WE may be either positive or negative (it may act as anode or cathode). The CE completes the circuit with the WE so that any current flowing through the WE is returned through the CE and vice versa. A second external circuit measures potential of the sensing electrode (SE) relative to a reference electrode (RE). As with the WE/CE electrode pair, the SE/RE electrode pair completes the circuit. The potentiostat is designed to measure SE potential while requiring virtually zero current between the RE and SE. The RE is often chosen to be a material that has a known potential at equilibrium conditions. By always maintaining negligible current flow through the RE, it can be kept at pseudo-equilibrium during measurements. The two external circuits therefore allow the potentiostat to measure the potential of the SE relative to the RE independent of the direction and magnitude of the current applied between the WE and CE.

**Fig. 1:**
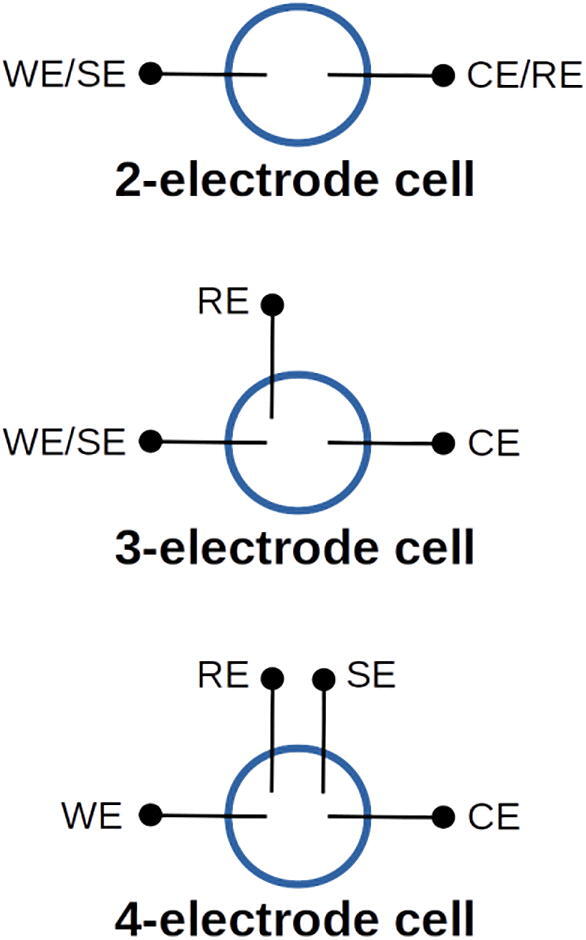
Illustration of three typical electrode configurations used in electrochemical cells.

The potentiostat has four leads that connect to WE, CE, RE, and SE in an external electrochemical cell. Depending on the type of measurement or experiment being carried out, the external cell will have one of three typical configurations, as illustrated in Fig. 1. In a 2-electrode cell, the leads for the WE and SE are shorted together and the leads for the CE and RE are shorted together. One electrode thus serves as both the WE and SE, while the other acts as both RE and CE. The potential measured across the 2-electrode cell includes the potential drop across electrodes, interfaces, and electrolyte solution separating the electrodes. In the two electrode configuration, current flows through the RE (because it is also acting as the CE). Interpreting the measured potential is difficult with a 2-electrode cell, and a more ubiquitous and less expensive DC power supply can be used instead of a potentiostat for many 2-electrode experiments. Most electrochemical measurements made with potentiostats are carried out using a 3-electrode configuration, in which the RE is placed separately at some location relatively near the WE. The WE also serves as the SE, allowing the potential of the WE to be measured relative to the RE without significant current passing through the RE. Since the RE is maintained at a known potential, the absolute potential of the WE is well characterized. The last configuration shown in Fig. 1 is the 4-electrode cell in which all four electrodes are separated from one another. While the 4-electrode cell is not commonly used, it does find some applications in systems where it is desired to measure the potential drop across some portion of the cell independent of the WE and CE.

A number of open-source potentiostat designs have been reported, with recent designs using advances in microelectronics to create compact, powerful instruments that rival the performance of comparable commercial instruments [2], [3], [4], [5], [6], [7], [8]. An open-source USB-controlled potentiostat/galvanostat was described by Dobbelaere, et al, that enables electrochemical cells to be controlled within potentiostatic limits of ±8 V and galvanostatic limits of ±25 mA, with data collected every 90 ms [2]. The instrument design by Dobbelaere is a significant advance over previously published open-source potentiostats, such as the CheapStat [7] and DStat [8] that are limited to ±1 V and do not provide galvanostatic control. The design also provides performance that is comparable to compact USB-controlled instruments sold commercially, as shown in Table 1. The hardware design, firmware, and control software are all completely open-source, allowing researchers to create customized measurement techniques by modifying hardware and software as needed. The cost of parts to build the Dobbelaere instrument is approximately $100, far less than the lowest cost commercial instrument. The Squidstat Solo manufactured by Admiral Instruments is currently advertised for sale at $1900. All other commercial instruments shown in Table 1 typically cost significantly more than the Squidstat Solo. The Dobbelaere instrument is also the only one that allows users the freedom to choose the open-source Linux operating system on the computer used for instrument control and data collection. As shown in Table 1, the most significant disadvantages of the Dobbelaere design are the lower maximum current range and the significantly slower maximum data collection rate. Modifications to the Dobbelaere design reported here begin to address some of these shortcomings. In addition, the printed circuit board layout was changed to facilitate placing it in an extruded aluminum enclosure with all required cable assemblies for electrodes, computer control, and power. The completed hardware build described here is thus a finished product, suitable for general electrochemical research and teaching in a laboratory setting.

**Table 1:** A comparison of key technical specifications of commercial instruments with the open-source design of Dobbelaere [2] and the modification of the Dobbelaere design reported here*.*

Instrument Name	Supplier	Maximum Current (mA)	Maximum Voltage (V)	Minimum Sample Time (ms)	Software Operating System
EmStat 3+	PalmSens BV	±100	±4 V	1	Windows, Android
WaveNowXV	Pine Research	±100	±10 V	0.5	Windows
Squidstat Solo	Admiral Instruments	±100	±10 V	1	Windows, OSX
Dobbelaere design	–	±25	±8 V	90	Windows, OSX, Linux
MYSTAT design (this report)	–	±200	±12 V	90	Windows, OSX, Linux

## 2 Hardware description

The goals of the new design were to (1) increase the current range and voltage range of the instrument, and (2) allow facile assembly of the instrument in an enclosure. The current and voltage ranges with the new design are significantly higher than comparable commercial instruments, as shown in Table 1. The larger current and voltage limits are particularly useful for broadening the application of the instrument. Higher current allows the use of larger working electrodes and the study of high current density processes, such as electrochemical energy storage. The higher voltage limits allow the study of electrochemistry in nonaqueous solutions and for processes such as electrophoretic deposition that require relatively large driving voltages. The dimensions of the circuit board were chosen to allow assembly in a commonly available extruded aluminum enclosure. End panels with necessary cutouts and labels were also designed from circuit board material. The end panels can be created at the same time as the main instrument circuit board and then attached to the extruded aluminum enclosure to provide a finished instrument without the need for any custom machining, labeling, or printing.

### 2.1 Instrument power supply

In the original design by Dobbelaere, et al, a computer USB port provided both power and communication to the instrument [2]. While this approach is convenient in that only a single cable is needed, it significantly limits the maximum power available. Dobbelaere used a charge pump to convert the 5 Volt power line from the USB connection into an on-board dual rail ±9 Volt supply, with maximum cell current limited to ±25 mA. To enable higher current and voltage, the charge pump was removed from the design. In its place, an external +15 Volt DC power supply (similar to that used to power laptops and other portable electronics) and a board mounted inverting power supply were selected to provide the majority of instrument power.

**Fig. 2:**
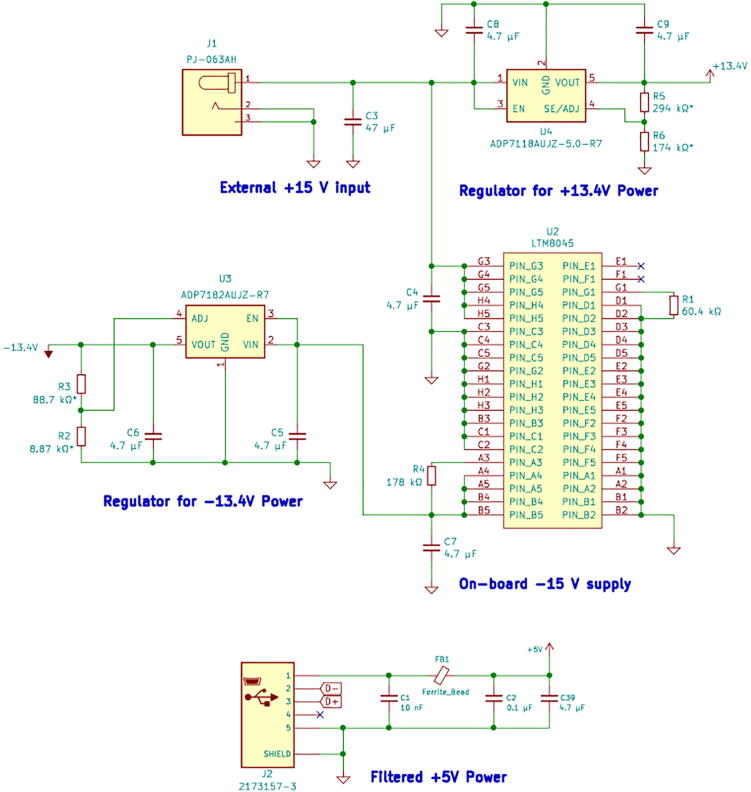
Schematic of instrument power supply circuitry.

Fig. 2 shows the circuit schematic of the power supply portion of the instrument design. The external 15 Volt DC supply is connected through a barrel jack. A 47 μF aluminum electrolytic capacitor was added for power input filtering and to avoid over-voltage transients observed with ceramic capacitor input filters [9]. An on-board inverting power supply (Analog Devices LTM8045) was selected to convert +15 Volt input into a −15 Volt power rail. The +15 Volt and −15 Volt rails are then passed through low dropout linear regulators that provide low-noise +13.4 Volt and −13.4 Volt lines, enabling ample overhead to apply ±10 Volts to the working electrode over the entire current range of the instrument. Power is also drawn from the USB connection to supply 5 Volts needed for several integrated circuits. Noise was first removed from the 5 Volt USB power line using a ferrite bead filter design based on an FTDI Application Note [10]. This noise-filtered 5 Volt line supplies power to the microcontroller, analog–digital converters, digital-analog converter, and a voltage reference.

### 2.2 Digital circuitry

**Fig. 3:**
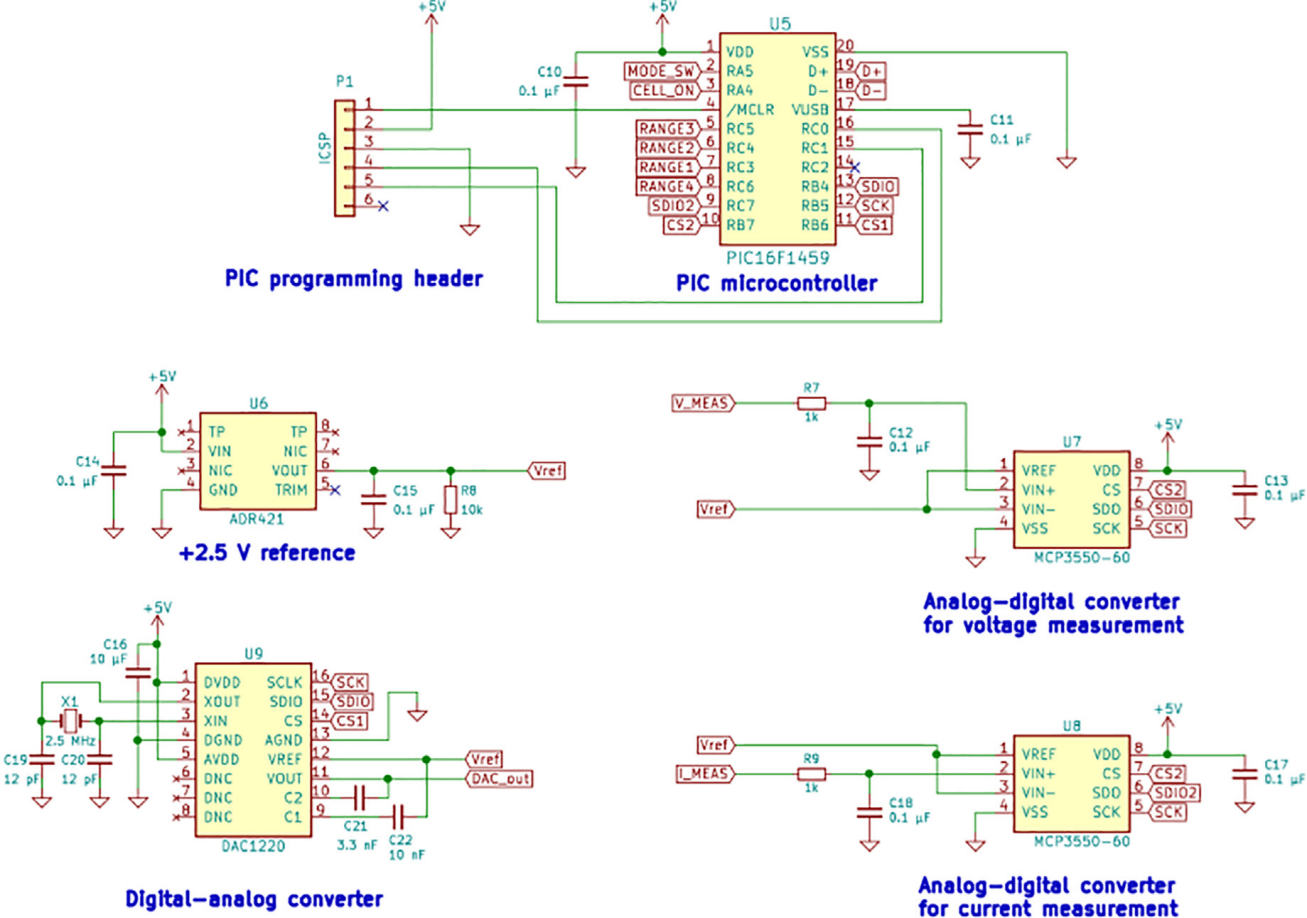
Schematic of digital circuitry.

Fig. 3 shows the schematic of the digital circuitry used in the instrument, which is essentially unchanged from the original design. The only significant change is the reassignment of input/output pins on the microcontroller to accommodate four current ranges instead of the original three. The microcontroller (Microchip Technology, PIC16F1459) input/output pins numbered 7, 6, 5, and 8 are used to control current ranges of ±200 mA, ±20 mA, ±0.2 mA, and ±0.002 mA, respectively. The digital-analog converter (Texas Instruments, DAC1220) provides 20-bit resolution. Therefore, the applied current resolution is ~380 nA in the highest current range and ~3.8 pA in the lowest current range. In potentiostatic mode the digital-analog converter provides applied voltage resolution of ~26 μV given the measured potential range of −13.4 V to +13.4 V. The 22-bit analog–digital converters (Microchip Technology, MCP3550-60) provide measured voltage resolution of ~6 μV, and measured current resolution between ~1 pA to ~100 nA, depending on the current range.

The sensitive direct-current measurements made by this instrument are susceptible to noise injected from the alternating current power mains. The model MCP3550-60 analog–digital converter was chosen because it has a built-in notch filter to remove 60 Hz signals, which is the frequency of alternating current power used in North America. Several other models of this analog–digital converter are available that are drop-in replacements. Model MCP3550-50 has a 50 Hz notch filter and should be chosen if that is the alternating current frequency of local power. Alternatively, model MCP3551 has both 50 Hz and 60 Hz notch filters and could be chosen for more universal application of the instrument. An instrument was built and tested using the analog–digital converter model MCP3553, which lacks a notch filter. It was found that noise was unacceptably high in measurements taken without a notch filter on the analog–digital converter.

### 2.3 Analog circuitry

**Fig. 4:**
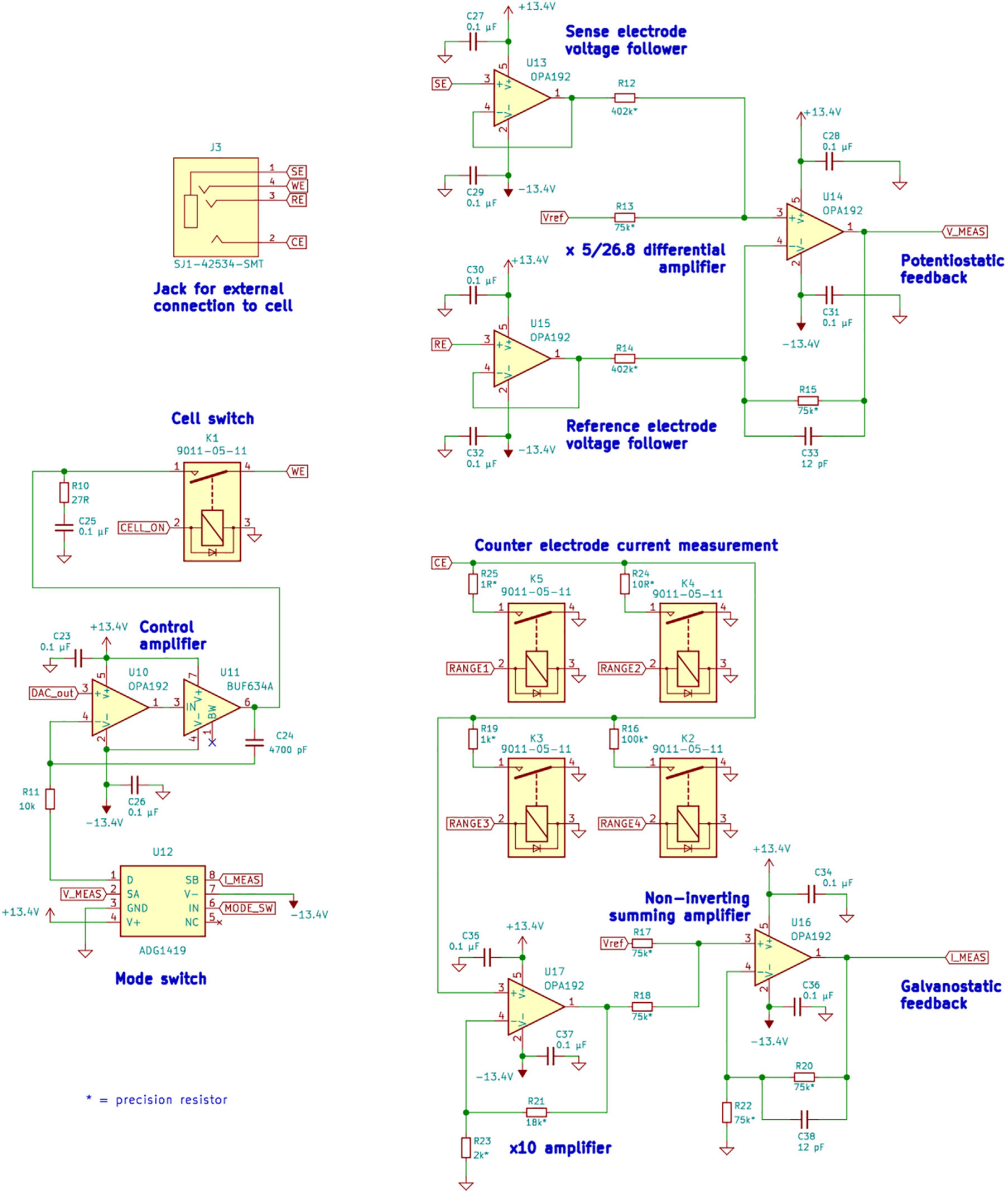
Schematic of analog circuitry.

The analog circuitry schematic is shown in Fig. 4. Several design changes were required for the analog circuitry in order to increase the maximum current range to ±200 mA. The original design included three current ranges: ±20 mA, ±0.2 mA, and ±0.002 mA. Precision resistors mapped each of these current ranges to an analog signal of 0–5 Volts that was used by the analog–digital converter for current measurement. In the new design, the three original current ranges were kept and a fourth range of ±200 mA was added. To enable use of this fourth current range, an additional reed relay and resistor were added to allow switching to the new higher current range and to provide the 0–5 Volt analog signal that is used to measure current. The firmware was modified to enable one of the originally unused microcontroller pins for the fourth current range. The original design used current output from the operational amplifier (Texas Instruments OPA4192) to supply the working electrode. Since the maximum output current of the operational amplifier is limited to ±65 mA, a buffer was added (Texas Instruments BUF634A) to boost the maximum rated output current to ±200 mA.

### 2.4 Design changes to facilitate assembly

The original potentiostat design resulted in an instrument on a bare 50 mm × 50 mm square circuit board rather than a finished device in an enclosure suitable for use on a lab bench [2]. The exposed circuit board can easily be damaged by mechanical contact with components or by spilling liquid solutions onto it. It was suggested by Dobbelaere that the instrument could be housed in any available small enclosure after making holes necessary for the USB cable and electrode leads. However, modification of an enclosure requires additional tools to construct. Feeding cables through an enclosure opening also makes swapping cables difficult or impossible without disassembly of the instrument.

**Fig. 5:**
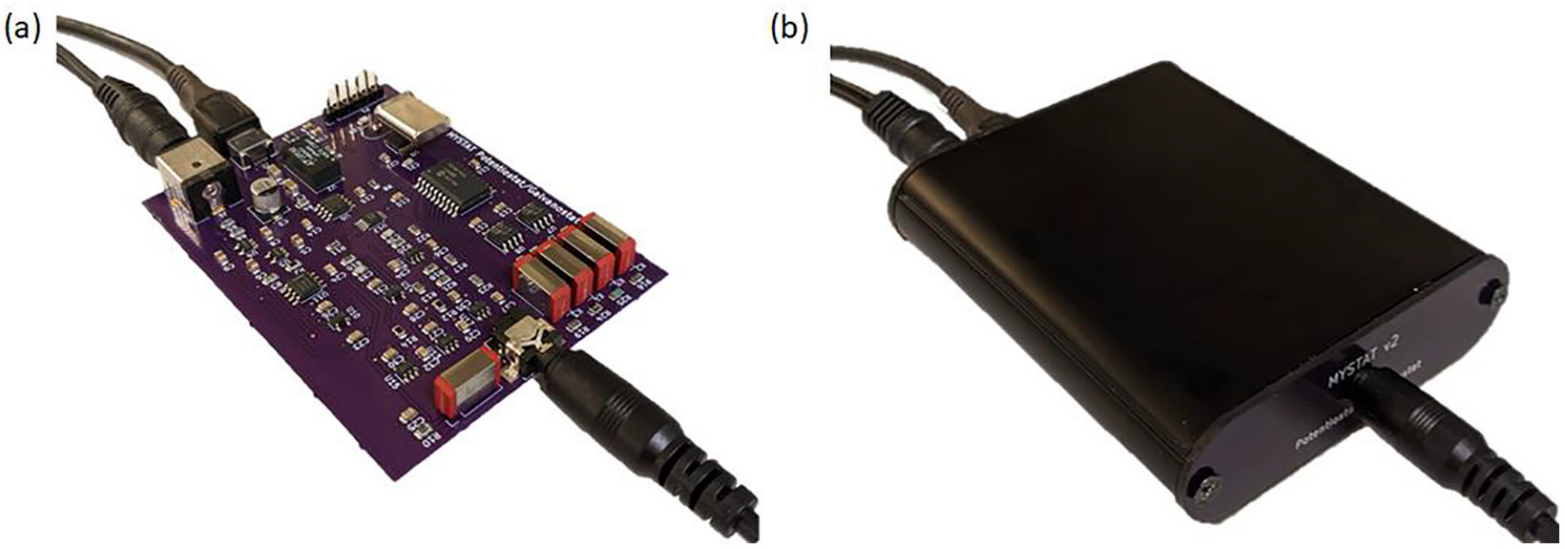
Photographs of the assembled instrument with (a) exposed circuit board and (b) within the enclosure.

Photographs of the main circuit board and the finished instrument are shown in Fig. 5. The overall dimensions of the circuit board were changed to 60 mm × 81 mm in order to allow it to fit into a commercially available extruded aluminum enclosure (Hammond Manufacturing, Model 1455B802). The extruded aluminum enclosure comes with solid plastic end plates that may be machined to provide openings for external power supply plug, USB connection, and the plug used to connect the electrode leads. However, to allow for easier assembly, “dummy” circuit boards were used instead of the plastic end plates. These end plates made from circuit board material can be manufactured at the same time as the main instrument circuit board. The circuit board end plates have the same dimensions as the plastic end plates, and have the required screw holes for attaching them to the aluminum case, required openings for the external data and power connections, and have silk screen printed labeling of the connections. The entire instrument can therefore be assembled without the need for any machining or printing. Other minor changes were made to fit the instrument into the compact enclosure. The mini-USB connector in the original design was replaced with a micro-USB connector to conserve space. The terminal block in the original design was replaced with a jack connector so that the four electrode leads can be connected to the instrument using a single plug rather than using screw connections to attach individual electrode leads directly to the circuit board. This open-source instrument is useful for:

• Voltammetry, amperometry, cyclic voltammetry, and charge/discharge measurements.
• Research and development of electrochemical materials and devices, such as batteries, supercapacitors, sensors, coatings, fuel cells, and electrocatalysts.
• Development of custom electrochemical measurement techniques.
• Building low-cost instrumentation to facilitate running parallel experiments and to provide equipment to students in teaching laboratories.

## 3 Design files

The design files are all contained in a single compressed zip archive named mystat_repository.zip provided at http://doi.org/10.5281/zenodo.4252476. Within the zip archive are three folders named control_software, firmware, and hardware. The control_software folder contains the Python source code for the software used to control the instrument, executable versions of the software for Windows and Linux operating systems, and a device driver that is required for Windows. The firmware folder contains source code for the microcontroller firmware as well as a compiled version of the firmware that may be used to program the microcontroller. The hardware folder contains KiCad design files for the circuit boards. KiCad is a free and open source cross platform electronics design suite that can be used to modify the circuit board design. Gerber files are also included in the hardware folder that may be used to fabricate the circuit boards. The bill of materials is provided in both csv and html format within the hardware folder.

Design files summary

**Table:** 

Design file name	File type	Open source license	Location of the file
*mystat_repository.zip*	Compressed archive containing source code and executable versions of control software and micro-controller firmware. Also contains bill of materials, KiCad files that may be used for modifying the circuit board design and Gerber files for fabricating circuit boards.	GPL-3.0	http://doi.org/10.5281/zenodo.4252476

## 4 Bill of materials

The bill of materials is included within the hardware folder of the archive provided at http://doi.org/10.5281/zenodo.4252476. Two versions of the bill of materials are given. The csv formatted file includes all required components, including the external 15 Volt power supply, aluminum enclosure, power cables, cell cables, and alligator clips to terminate the cell cables. The total cost of this bill of materials is $201.05. Printed circuit boards must also be produced. The cost of purchasing boards from OSH Park is $25.17 each in low quantities, making the total cost of one instrument $226.22. The second version of the bill of materials is provided in html format that can be opened in a web browser. The html file contains only those components that need to be placed on the printed circuit board. The html file is useful to refer to during assembly because it has an illustration of the location of each component on the circuit board.

## 5 Build instructions

### 5.1 Procurement of electrical components

The electronic components, cables, connectors, and extruded aluminum enclosure are standard parts that may be purchased from a number of retailers, such as Mouser, Digikey, or Newark. If any of the specific passive components listed in the bill of materials are out of stock, they may be substituted with equivalent rated parts. If components are substituted, ensure that the substitute has the same footprint and similar or higher ratings for voltage and power dissipation.

### 5.2 Printed circuit board and stencil manufacturing

The printed circuit boards can be obtained in small quantities at relatively low-cost by any number of manufacturers specializing in prototyping, such as OSH Park, Screaming Circuits, Seeed Studio, and Sunstone Circuits, among others. Gerber files are provided to facilitate circuit board fabrication. Many circuit board manufacturers will provide instant price quotes upon receiving Gerber files. The main instrument board has 4-layers, with signal traces on the top and bottom layers and solid ground planes on the two internal layers. Although typically much more expensive than purchasing a bare PCB, some prototyping manufacturers will provide complete assembly of the finished circuit board with the components listed in the supplied bill of materials. A pick and place file for automated assembly can be generated by opening the included PCB design in KiCad and selecting: File → Fabrication Outputs → Footprint Position (.pos) File. For someone without prior experience, manual assembly as described below will likely take at least 2 to 3 h.

For manual assembly of components, it is recommended that a stencil be purchased to facilitate application of solder paste onto the circuit board. Manufacturers can produce a stencil using the included Gerber file for the front paste layer. Stencils are constructed from either polyimide or stainless steel, and both types work well. Polyimide stencils are less expensive, while stainless steel stencils are more durable. Additional Gerber files are provided for the front and back end panels of the instrument enclosure. It is recommended that the end panel boards be manufactured at the same time as the main circuit board. These “dummy” two-layer boards are only used to cover the ends of the enclosure and provide labeled openings for power and signal connections. To save cost, the plastic end panels that come with the extruded aluminum case may be used instead of circuit board material. However, holes must be cut into the plastic. Crude holes may be made with a rotary cutting tool. Precise holes in the plastic end panels can be made using a laser cutter, with the included Gerber files as a guide.

### 5.3 Assembly and soldering

The majority of the parts that must be soldered to the board are surface mount components, which should be soldered before the through-hole components. It is recommended that eutectic tin/lead solder paste (63% tin, 37% lead) be used for surface mount components. Alternatively, lead-free pastes are available that are less harmful to the environment, but more difficult to solder. The solder paste is easiest to apply using a stencil when the paste is at room temperature. The stencil is first aligned on the board and held in place using tape. Then a squeegee (a plastic credit card is a good alternative) is used to spread the paste. After removing the stencil, each component must be placed on the board manually.

A bill of materials is provided as an html file that may be opened in any web browser. This html file highlights the locations of components on the board and is very useful to refer to during assembly. Great care must be taken to avoid smearing the paste while placing parts. Manual assembly requires a steady hand and good eyesight. It is recommended that an inexpensive USB microscope or magnifying glasses be used to guide positioning of components. Fine-tipped forceps may be used to pick up components and place them on the board. A vacuum pen may also be used for positioning components on the board. After all surface mount parts are placed, the board must be heated to solder the components to the board. It is recommended that an oven designed specifically for reflow soldering be used. A reflow oven can be programmed to precisely follow the temperature versus time profile specified by the solder paste manufacturer. There are a variety of lower-cost reflow soldering options available, such as using a toaster oven, hot plate, or electric skillet [11]. After reflow soldering, the remaining through-hole components are attached manually using a soldering iron. The crystal oscillator must be hand soldered with ~90 degree bend in the leads (see Fig. 5a). Otherwise, the height of the crystal will prevent the board from fitting inside the enclosure.

### 5.4 Circuit board inspection and initial electrical testing

After assembly, it is recommended that the board be inspected carefully using a USB microscope or magnifying glasses. Check that all components are in their proper position and that there are no obvious visible problems. Excess solder that may cause shorts can sometimes be removed with a solder wick. A hot air rework station can sometimes be used to reposition components. If there are numerous or severe problems with soldering, the board may need to be redone completely using new parts. If there are no visible problems, power should then be applied to check for electrical shorts. Both the 15 Volt external supply and 5 Volt supply from the USB cable connections should be made. It is recommended to use a powered USB hub for testing rather than connecting the instrument directly to a computer. If there is an electrical short on the 5 Volt line, it may cause damage to a computer’s USB port. An electrical short will likely cause the board to become hot in places. This is easily detectable using a thermal imaging camera. A bench top power supply that has adjustable voltage and current limits may also be used to safely test for short circuiting. By setting a low current limit, the adjustable power supply would minimize the chance of board overheating in the case of a short circuit.

### 5.5 Programming the microcontroller and placing in an enclosure

The next step in assembly is programming the microcontroller with firmware. Programming requires the purchase of a low-cost programming device (PICKit3 is recommended), available from a variety of retailers. The programming software required is free to download for Windows, Linux, and MacOS operating systems from Microchip Technology as part of the MPLAB X Integrated Development Environment. The programmer should be connected from the computer to the programming header on the potentiostat circuit board. The micro-USB port on the potentiostat should also be connected to the computer in order to provide 5 Volt power to the microcontroller. It is not necessary to connect the 15 Volt power supply to the potentiostat during programming. Follow the MPLAB X Integrated Development Environment software instructions to program the microcontroller using the firmware supplied, which has the file name “firmware.hex.”

After programming the microcontroller, the board is placed into the extruded aluminum enclosure. The two endplates are then attached using screws. The last part of assembly is fabricating the electrode leads. A recommended list of parts for the electrode leads is given in the bill of materials. If the specified four-wire cable assembly is used, the wire colors will correspond to the following: WE (green), CE (red), RE (white), SE (black). The electrode cable should be inserted into the jack connector when the instrument is disconnected from electric power to prevent shorts between electrodes. The electrode jack connector should never be inserted or removed while the instrument is powered on, as there is a risk of short circuits forming during insertion or removal of the plug.

### 5.6 Preparation of a computer for controlling the instrument

A computer with a USB connection is needed to control the instrument and record data. It should be possible to use the Windows, Linux, or MacOS operating system. The instrument has been tested using several different laptops and tablets running Windows 10 and Debian Linux. No testing was done using MacOS. A powerful computer is not needed. The inexpensive Raspberry Pi 4 was found to be capable of controlling the instrument. The instrument can also be controlled remotely over a wireless network using virtual network computing (VNC) connection. The Raspberry Pi operating system includes VNC software that makes setting up remote instrument control straightforward [12].

For the Windows operating system, a driver must be installed. The open-source Zadig program has been included in the repository of files to facilitate installation of the driver. Plug the USB cable from the instrument into the Windows computer, then run Zadig. The instrument will appear in the device list as “USB Potentiostat/Galvanostat”. Select the “libusbK” driver from the drop down list and click to install. On the Linux operating system, driver installation is not needed. To allow device access on Linux without requiring root permissions, make sure that the user is a member of the “plugdev” group, and create a text file “/etc/udev/rules.d/99-mystat.rules” that contains the line:

SUBSYSTEM==“usb”, ATTRS{idVendor}==“a0a0″, ATTRS{idProduct}==”0002″, GROUP=“plugdev”, MODE=“0666″

The file containing the above line of text will need to be edited and saved using root permissions.

After enabling device access on the computer as described in the previous paragraph, the instrument control software can be run. Single executable files have been provided for Windows and Linux operating systems. For Windows, save the file named “mystat.exe” from the repository to the computer and start it by double clicking. For Linux, save the file named “mystat” to the computer. Make sure this file is executable by issuing the command “chmod + x mystat”, then run the program with the command “./mystat”. The Python source code for the control software has also been provided in the file named “mystat.py”. The software can also be run from the source code if Python and the required libraries and modules have been installed on the computer.

## 6 Operation instructions

Operation of the instrument is largely unchanged from the design published by Dobbelaere, et al [2]. The minor procedural differences arise as a result of the expanded current and potential ranges. An overview of these procedural differences is described.

### 6.1 Calibration

The calibration procedure described in section 6.3 of the Dobbelaere, et al paper is suitable for calibrating the MYSTAT with slight adjustment. The reader is referred to the Dobbelaere paper for calibration of potential offset, current offset, DAC offset, and DAC gain as there are no procedural differences between the two instruments. Calibration of shunt resistor values requires some adjustment due to the presence of an additional shunt resistor for the 200 mA current range. In the original design, R1, R2, and R3 correspond to the 20 mA, 200 μA, and 2 μA current ranges respectively [2]. In the modified design, R1, R2, R3, and R4 correspond to the 200 mA, 20 mA, 200 μA, and 2 μA current ranges respectively. Note that the presence of the additional 200 mA current range alters the associated shunt resistor number assignments. Unlike the original design in which only R1 required calibration, in the modified design both R1 and R2 require calibration and will likely need to be set to a value greater than 1.0000. R3 and R4 should both be set to 1.0000 and should not require significant adjustment, if any, as long as precise shunt resistors were used in device assembly.

While adjusting R1, the instrument should be set to the 200 mA current range and while adjusting R2, the instrument should be using the 20 mA current range. The current range being used is displayed just above the real-time plot, as shown in the screenshots provided by Dobbelaere, et al [2]. The following procedure, slightly modified from the procedure described by Dobbelaere, et al, is recommended for calibration of the shunt resistors:

• Attach WE and SE to one leg of an accurate 1.000 kΩ resistor and attach RE and CE to the other leg. Using the 200 mA current range, put the instrument in potentiostatic mode, and set a potential of 7.000 V. Turn the cell on and adjust R1 until current is exactly 7.00 mA. Pressing “Save to device” saves the value.
• Set the current range to 20 mA then adjust R2 until it is exactly 7.000 mA. Pressing “Save to device” saves the value.

### 6.2 Measurement ranges for current and voltage

For low current values, the instrument is capable of outputting potentials as high as ±12 V. Such values may be set in potentiostatic mode for the 20 mA, 200 μA, and 2 μA current ranges and are attainable in the 200 mA current range if current is low. The maximum output voltage decreases as output current is increased due to the power supply headroom required by the buffer amplifier (see the data sheet for Texas Instruments BUF634A). The required headroom for the buffer increases with increasing output current. At the highest allowable current of ±200 mA, the maximum output voltage is reduced to approximately ±10 V. Therefore, voltage settings should be limited to ±10 V if it is required that output voltage not drop over the entire current range of the instrument. It is not recommended that potential ever exceed ±12 V. It is also advised that current not exceed the upper bounds of the associated current range as this may result in damage to the device, especially if values greater than 200 mA are achieved. To prevent damage to several of the instrument’s components, an emergency shutdown feature was added to the Python code such that if current exceeds ± 220 mA, current will automatically be reset to zero. The circuit board was designed with numerous vias to solid copper planes that act both as electrical ground planes and to disperse heat throughout the board. However, the instrument design reported here does not provide for active cooling using fans, so it is likely that the circuit board temperature will increase notably if high output current is used for extended time. Extreme temperatures can adversely affect electrical performance or permanently damage some components. No such thermal damage or performance issues were observed in any of the instrument testing. However, if the desired application of this instrument calls for maintaining the output near the maximum operating limits of voltage and current for long periods of time, the user is encouraged to monitor board temperature during initial testing. If needed, active cooling can be added. It would be trivial to add a 2-wire 5 V DC cooling fan powered by connection to pins number 2 and 3 of the PIC programming header on the board. The enclosure would need to be modified to allow air flow if a cooling fan is to be added.

## 7 Validation and characterization

### 7.1 Instrument noise measurements

For potentiostatic mode noise measurement, the WE and SE lead pair and the RE and CE lead pair were each connected together. With an open circuit maintained between these two joined lead pairs, the instrument was placed in potentiostat mode. The output voltage was set to zero and the measured current was recorded. The standard deviation of measured current was calculated based on 1000 measurements in each of the four current ranges. For the 200 mA, 20 mA, 200 μA, and 2 μA current ranges, the standard deviation in measured current was 383 nA, 46 nA, 515 pA, and 12 pA, respectively.

For galvanostatic mode noise measurement, the WE and SE leads were attached to one leg of a resistor, and the CE and RE leads were attached to the other leg of the resistor. The resistance used was 56 Ω for the 200 mA current range, 600 Ω for the 20 mA and 200 μA current ranges, and 5.9 MΩ for the 2 μA range. The instrument was placed in galvanostatic mode and the output current was set to zero. The measured voltage was recorded. The standard deviation of measured voltage was calculated based on 1000 measurements in each of the four current ranges. The standard deviation of the measured voltage was 29 μV, 32 μV, 18 μV, and 37 μV for the 200 mA, 20 mA, 200 μA, and 2 μA current ranges, respectively.

### 7.2 Ohm’s Law tests

**Fig. 6:**
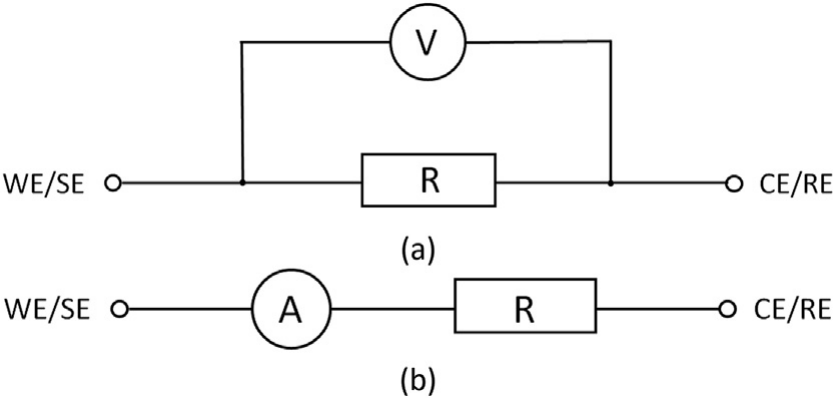
Circuit design for verification of potential and current measurement accuracy. (a) Circuit convenient for testing the 200 mA, 20 mA, and 200 μA current ranges, (b) Circuit convenient for testing the 2 μA current range.

To verify the accuracy of the instrument in all four current ranges, the device was calibrated and several different resistance values were used to make basic voltage and current measurements. Testing was carried out over the entire current and voltage limits of the instrument. WE and SE were attached to one leg of the resistor and CE and RE were attached to the other leg. A digital multimeter was hooked up in parallel and was used to verify the voltage values reported by the instrument. The circuit design is shown in Fig. 6a. This setup worked well for testing the 200 mA, 20 mA, and 200 μA current ranges. A 56 Ω resistor was used for testing the 200 mA range and a 600 Ω resistor was used for testing the 20 mA and 200 μA current ranges.

When testing the 2 μA current range, a resistor with high resistance is needed to keep current low. A 5.9 MΩ resistor was used for this current range. Such resistance is high enough that with the setup in Fig. 6a, the multimeter draws significant enough current to affect measurements. To verify current measurements, the multimeter was placed in series as shown in Fig. 6b and used to measure current directly.

The instrument was tested using both potentiostatic mode by setting voltage and galvanostatic mode by setting current in all 4 current ranges. Since the multimeter is only capable of measuring voltage or current at any given time, Ohm’s Law, given by V = IR, was used to calculate the remaining parameter where V is voltage, I is current and R is resistance.

**Fig. 7:**
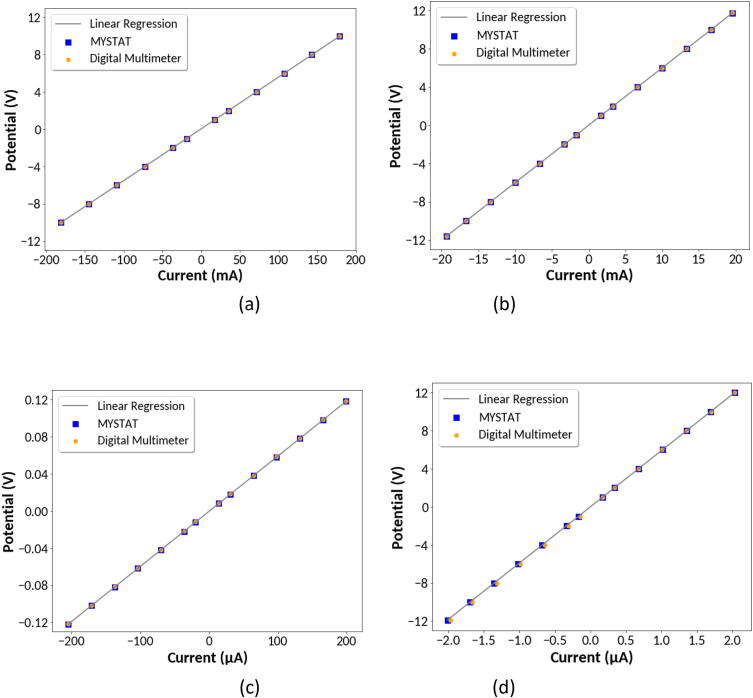
Comparison of voltage and current measurements to those from a digital multimeter using the (a) 200 mA (b) 20 mA (c) 200 μA and (d) 2 μA current ranges.

In Fig. 7, the data obtained with the instrument are compared to values obtained using the digital multimeter and Ohm’s Law. A regression line for the data obtained with the MYSTAT is also plotted. The data collected from the instrument agree with data obtained with the digital multimeter and values calculated using Ohm’s Law. It is possible that the MYSTAT is more accurate than the digital multimeter in the lowest 2 μA current range based on the linear voltage/current relationship. The R^2^ value for a linear fit to the MYSTAT data is 0.999999 while it is slightly less (0.999949) when using the multimeter data. The maximum measured current difference between the multimeter and the MYSTAT in the 2 μA current range was 41 nanoamps, and the mean difference was 18 nanoamps. The maximum measured voltage difference between the MYSTAT and digital multimeter from the 200 μA current range data was 0.4 mV with a mean value of 0.35 mV. For the 20 mA range, the maximum measured voltage difference was 14 mV with a mean difference of 5.6 mV. For the 200 mA range, the maximum measured voltage difference was 10 mV with a mean difference of 4.7 mV. To further evaluate the obtained data, linear regression was performed on the MYSTAT measurements. According to Ohm’s Law, the slope of the regression line should be equal to the resistance of the resistor and the y-intercept should be close to zero. The linear regression equations for each of the four current ranges are given in Table 2. The calculated resistance values are all within the reported ± 1% tolerance for the resistors [13], [14], [15].

**Table 2:** Linear regression equations for Ohm’s Law data obtained with the MYSTAT.

Current Range	Linear Regression	R^2^ Value
200 mA	V = (55.5 Ω) I + 0.0350 V	0.999987
20 mA	V = (600 Ω) I + 0.0000450 V	0.999999
200 µA	V = (594 Ω) I + 0.0003798 V	0.999999
2 µA	V = (5,909,000 Ω) I + 0.0008180 V	0.999999

### 7.3 Cyclic voltammetry measurements

**Fig. 8:**
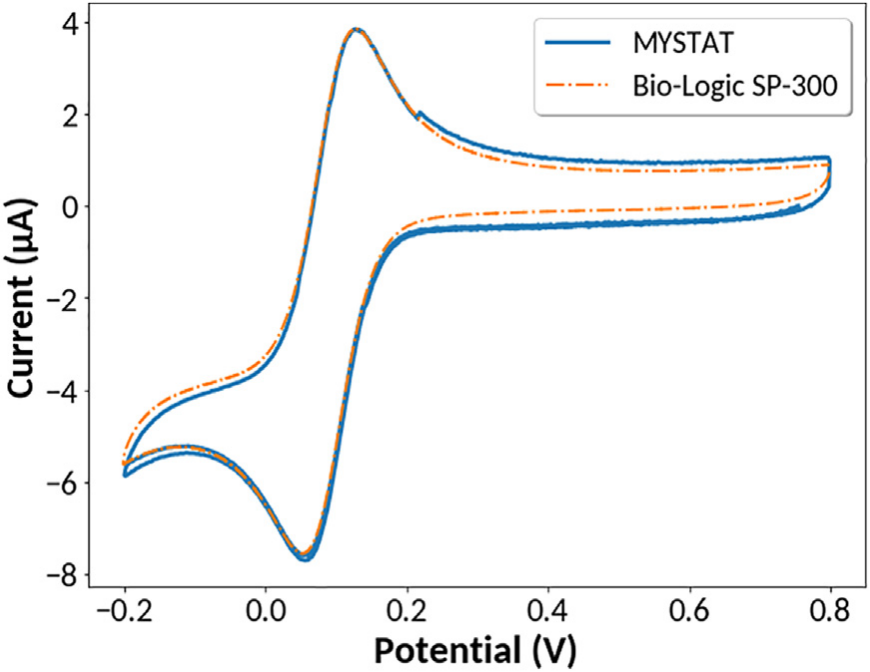
Cyclic voltammetry of potassium hexacyanoferrate (III).

The instrument’s ability to perform cyclic voltammetry was tested with an aqueous, 5 mM solution of potassium hexacyanoferrate (III) in 1 M potassium nitrate. A Compact Voltammetry Cell Kit was purchased from Pine Research. The kit includes several types of electrodes, of which the platinum electrode on ceramic was used. Cyclic voltammetry was performed with a scan rate of 5 mV/sec from −0.2 V to 0.8 V. As a comparison, an identical scan was performed using a Bio-Logic SP-300 commercial potentiostat. The data are plotted in Fig. 8.

**Fig. 9:**
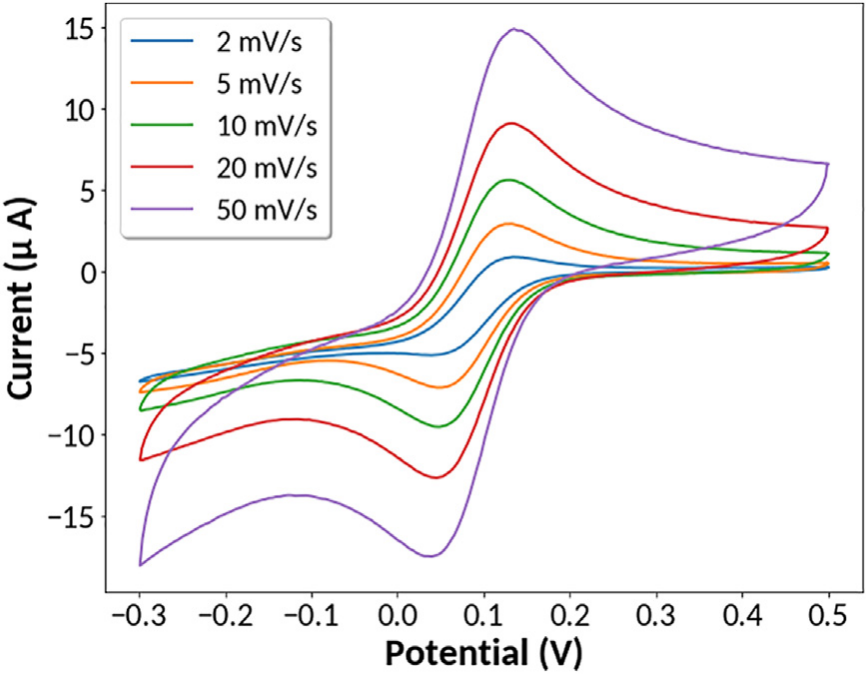
Cyclic voltammetry of potassium hexacyanoferrate (III) at various scan rates.

The data obtained with the MYSTAT closely align with data obtained with the Bio-Logic SP-300 as shown in Fig. 8. Both instruments recorded an anodic peak potential of 0.13 V and a cathodic peak potential of 0.05 V. For the data presented in Fig. 8, the maximum difference in measured values between the two instruments is 0.42 μA with a mean difference of 0.18 μA. One notable discrepancy between the data obtained is the 0.14 μA difference in current at potentials greater than 0.22 V. This jump occurs when the current range transitions from the 200 μA current range to the 2 μA current range. Depending on the scan rate, this discrepancy may be more dramatic and produce undesired bumps in the data caused by the instrument repeatedly changing between current ranges. This effect may be prevented by disabling the lower of the two current ranges under the “Autoranging” section of the “CV” tab. To further demonstrate the instrument’s capacity to perform cyclic voltammetry, several scans were performed at 2, 5, 10, 20, and 50 mV/sec. Measurements were performed from −0.3 V to 0.5 V with the 2 μA current range disabled. As expected, an increase in scan rate results in increased peak cathodic and anodic current values as well as an increase in peak-to-peak separation as shown in Fig. 9.

### 7.4 Battery testing

**Fig. 10:**
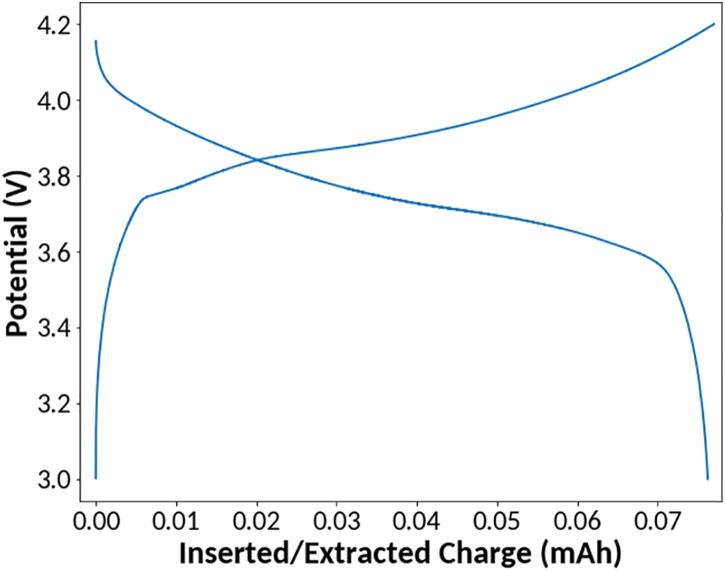
Charge/Discharge of 3.7 V 80 mAh Li-Ion battery.

The instrument’s ability to perform charge/discharge measurements was tested with a 3.7 V 80 mAh Li-Ion battery purchased from Renata (Manufacturer Part Number: ICP501022UPM). Charge/discharge measurements were performed from 3 V to 4.2 V with a charge current of 40 mA and discharge current of −40 mA. Potential is plotted against inserted/extracted charge in Fig. 10. The measured charge capacity of 77.01 mAh and discharge capacity of 76.25 mAh are within the ±5 mAh tolerance reported on the battery datasheet, demonstrating reasonable data output for performing charge/discharge measurements [16]. The expanded current range allows for these types of high current measurements comparable to those of a commercial potentiostat.

### 7.5 Ultracapacitor testing

**Fig. 11:**
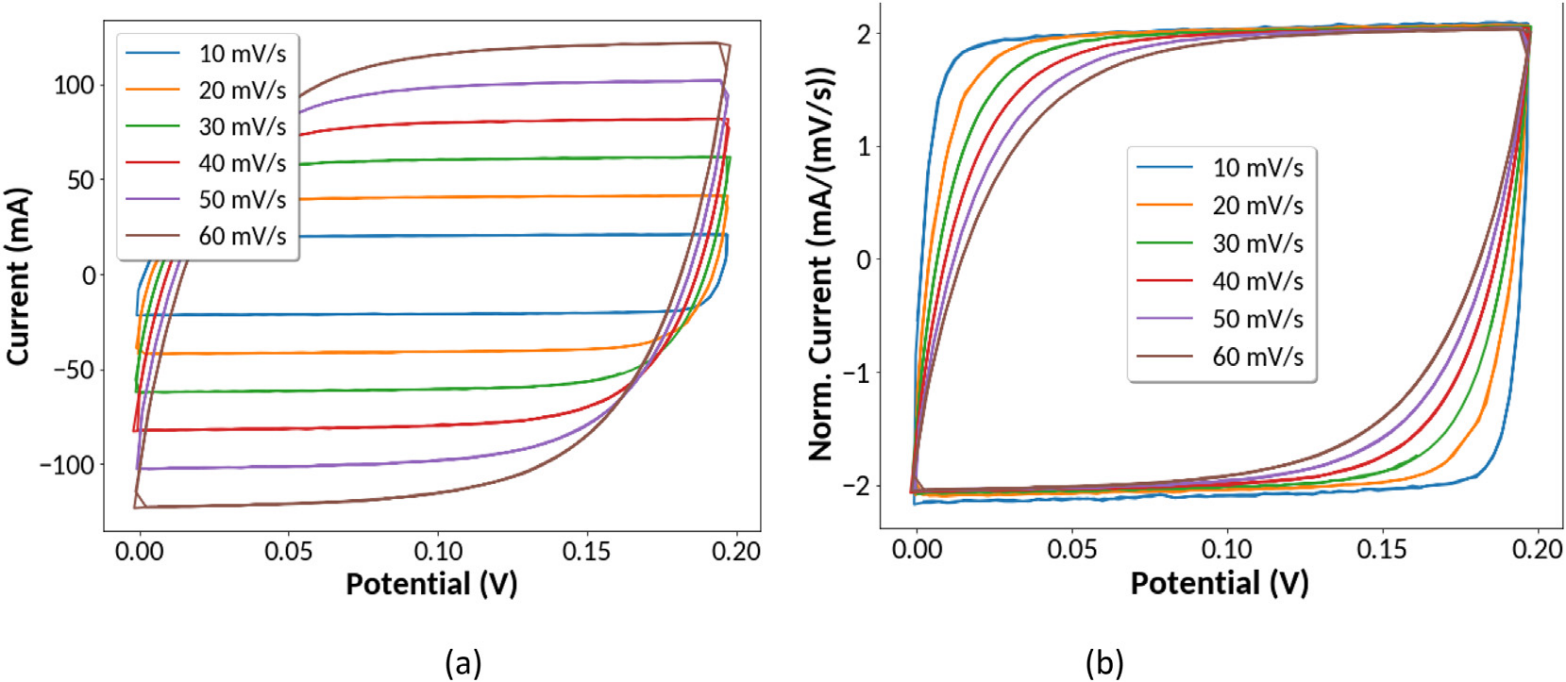
Cyclic voltammetry measurements on a commercial ultracapacitor rated at 2.5 Farads using scan rates from 10 to 60 mV/s. (a) Measured current in mA versus voltage. (b) Measured current in mA normalized by the scan rate in mV/s.

To demonstrate high current and voltage capabilities of the instrument, tests were carried out on a commercial electrical double layer ultracapacitor (Tecate Group, model 17–0106-0001) having a rated 2.5 Farad capacitance. Fig. 11 shows data from cyclic voltammetry scans. For an ideal capacitor, the charge/discharge current is equal to the capacitance multiplied by the change in voltage with time. Therefore, the current should be proportional to the scan rate set for cyclic voltammetry. Absent any kinetic limitations, a perfect capacitor would therefore result in a rectangular shaped plot from a cyclic voltammetry measurement. Fig. 11(a) shows the measured current in mA from cyclic voltammetry using scan rates from 10 mV/s to 60 mV/s. In Fig. 11(b) the measured current in mA has been normalized to the scan rate in mV/s. The results show that the plots deviate from the ideal rectangular shape due to kinetic effects. The deviation increases with increasing scan rate as expected. The horizontal portion of the plot in Fig. 11(b) should be equal to the capacitance in Farads. Despite being rated as a 2.5 Farad capacitor, the data indicate the actual capacitance in closer to 2 Farads in this low voltage window used for cyclic voltammetry.

**Fig. 12:**
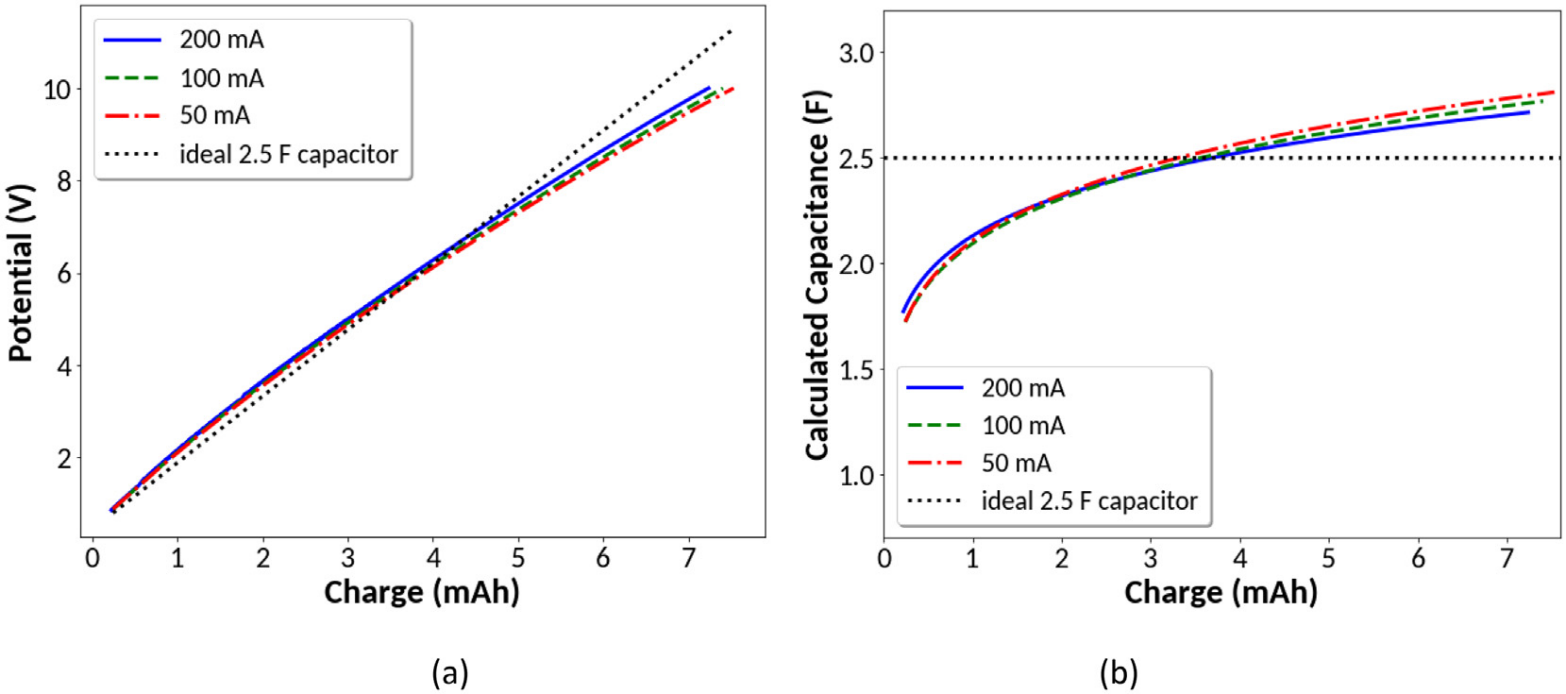
Galvanostatic charging of a 2.5 Farad ultracapacitor using charging currents of 50 mA, 100 mA, and 200 mA. (a) Measured voltage versus total inserted charge. (b) Calculated capacitance versus total inserted charge.

The ultracapacitor selected for testing has a rated maximum voltage of 10.8 V. The capacitor was charged from 0.5 to 10 Volts using constant applied current of 50 mA, 100 mA and 200 mA. Fig. 12(a) shows the voltage of the capacitor versus inserted charge in mAh. Absent kinetic effects, an ideal capacitor should display a straight line on this plot with the slope being proportional to capacitance. The data show a slight curvature indicating the device does not behave as would be expected for a perfect capacitor. This slight nonlinear voltage/current behavior is typical of electrical double layer capacitors charged galvanostatically and may be represented by [17]:

(1) $$V\left(t\right)=V_{0}+I_{\mathrm{CC}}\left(R_{S}+\frac{t^{\alpha }}{Q\Gamma \left(\text{1}+\alpha \right)}\right)$$

where V(t) is voltage versus time, V_0_ is the initial voltage, I_CC_ is the charging current, R_S_ is the series resistance of the capacitor, Q is a pseudo-capacitance, α is a dispersion coefficient, and Γ(x) is the Euler Gamma Function of x. The charging data were fit to the above function using R_S_ = 0.400 Ω from the series resistance specified by the manufacturer, and with Q and α as adjustable parameters. The fit to the data was then used to calculate the measured capacitance in Farads versus total inserted charge at the different galvanostatic charging currents, as shown in Fig. 12(b). The data show that the effective capacitance increases with charging time when charged galvanostatically. The effective capacitance is initially below the rated 2.5 Farads and increases above the rated capacitance. The mean capacitance calculated from the data in Fig. 12(b) is 2.49F, 2.46F, and 2.40F for the 50 mA, 100 mA, and 200 mA charging current, respectively. The calculated capacitance is close to the nominal rated value specified by the manufacturer, and the kinetic response during galvanostatic charging is consistent with reported measurements from other double layer capacitors [17]. The data from the ultracapacitor testing also demonstrate the utility of the instrument providing galvanostatic as well potentiostatic control over higher current and voltage limits in comparison to previously published open source designs.

### 7.6 Use of the instrument for teaching

The relatively low cost and compact size of the instrument makes it potentially useful for teaching laboratories to allow students to learn about electrochemical measurements through hands-on experiments. Standard DC electrochemical techniques can be used to measure concentrations of ions or molecules in solution, rates of corrosion, and capacity of batteries. In addition, a variety of simple sensors involve the application and measurement of current and voltage. In these sensor applications, the MYSTAT can serve as a very accurate constant current or constant voltage source for excitation of a sensor, and provide accurate current or voltage readings from a sensor. A few low cost sensors were tested to show how the MYSTAT instrument can be used to teach some basic concepts and show how things like pH, temperature, and weight are measured electronically.

**Fig. 13:**
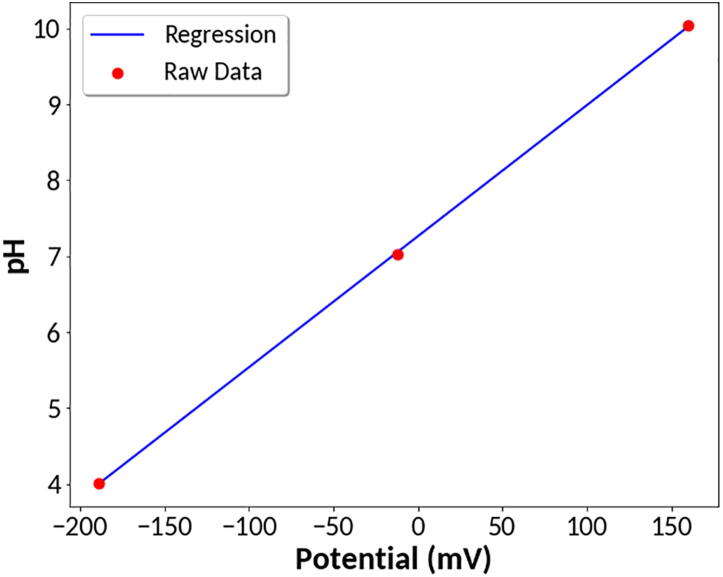
pH calibration curve.

*pH measurement:* A pH meter works by measuring the electric potential of an H^+^ ion selective electrode relative to a reference electrode. The electric potential of the pH electrode changes linearly with pH. After calibration with solutions of known pH, the pH meter converts the measured electric potential to a pH reading. To demonstrate this, an Extech 601,500 standard pH electrode was purchased and its potential was measured using the MYSTAT. The pH electrode is terminated with a BNC connector, so an adapter was used to convert the BNC connector to two test leads. The MYSTAT was then connected to the pH electrode with the WE and SE pair attached to one lead and the CE and RE pair attached to the other lead. The open circuit potential of the electrode was measured in several BioPharm Buffer Reference Solutions of known pH. The linear relationship between measured electrode voltage and pH is shown in Fig. 13. A linear fit to the data provides a calibration curve that can be used to convert measured potential into a pH reading. To test the calibration curve’s accuracy, a 0.1 M acetate buffer was prepared and measured to have a pH of 4.58 with an Apera Instruments PH700 Benchtop pH Meter. A voltage of −155 mV was measured for the same buffer solution using the potentiostat with the Extech 601,500 electrode. According to the calibration curve, the measured potential corresponds to a pH of 4.59. The results demonstrate that the MYSTAT is capable of pH measurement accuracy equivalent to that of a standard laboratory pH meter.

*Temperature measurement* One method for precisely measuring temperature electronically is using a resistance temperature detector (RTD). The temperature measurement is based on the change in electrical resistance of a metal with temperature. The most common RTD probes use a platinum element having a precise resistance of 100 Ω at zero degrees Celsius. The resistance increases nearly linearly with increasing temperature, with an increase of approximately 0.39 Ω per degree Celsius. An RTD temperature meter passes a small fixed current, usually around 1 mA, through the platinum element and measures the voltage drop across the element. The platinum resistance is then calculated using Ohm’s Law.

To demonstrate RTD temperature measurement, an inexpensive RTD probe was purchased (Adafruit PT100 model 3290). The probe is terminated in three leads. Two leads are colored red, and one is blue. The 3-lead RTD probe is designed for current to be passed between the blue lead and one of the red leads, while voltage is measured between the blue lead and the other red lead. The SE lead from the potentiostat was attached to one of the red terminals of the RTD. The WE connection was attached to the other RTD red terminal. The CE and RE connections were attached to the RTD blue terminal. The MYSTAT was placed in galvanostatic mode and a fixed current of 1 mA was passed through the RTD element while measuring voltage. The RTD probe was equilibrated in water baths having various temperatures as measured by a glass bulb thermometer, and the resistance of the RTD element was determined, as shown in Table 3.

**Table 3:** Measured resistance of a platinum RTD probe element compared to the expected value from the ITS-90 temperature standard.

Temperature (°C)	Measured Resistance (Ω)	Expected Resistance (Ω)
5	102	101.95
10	104	103.90
15	106	105.85
20	108	107.79
30	112	111.67
35	114	113.61
40	116	115.54

The data show that the MYSTAT instrument is capable of measuring temperature with an RTD probe. The voltage measurements were taken from the display on the potentiostat control software, which only gives values out to three decimal places (millivolts). As a result, the measured resistance was only accurate out to single Ohm level. The implied temperature accuracy is only within a ~2.5  °C range. However, the MYSTAT noise measurements show that the instrument is capable of much higher accuracy in voltage measurement. Assuming that voltage could be measured within 50 μV, the RTD element resistance would be accurate within 0.05 Ω. By recording a number of raw data voltage readings from the instrument and averaging them, the MYSTAT should be capable of very high accuracy temperature measurements using an RTD probe.

*Weight measurements* A load cell is a device that converts an applied force to some type of electrical signal that can be measured. A variety of different load cell types are available, but a low cost strain gauge load cell (Yohii model DC5-10 V) with a 300 g measuring capacity was used for demonstration. A strain gauge is a thin foil resistor whose resistance increases when placed under tension and decreases under compression. The strain gauge load cell is a hollow aluminum bar with four identical strain gauges arranged in a Wheatstone bridge configuration. When unloaded, the voltage drop between each leg of the Wheatstone bridge is zero. The aluminum bar is mounted so that it is fixed on one end and a load is placed on the other end. The applied load causes the bar to bend slightly, altering the resistance of the strain gauges. When the Wheatstone bridge is excited by an applied voltage or current, the applied force can be correlated to measured changes in voltage between the two legs of the Wheatstone bridge.

**Fig. 14:**
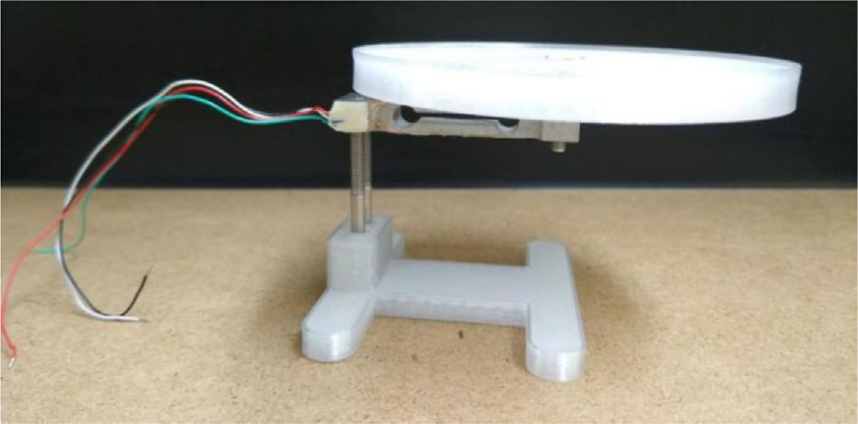
A 3D printed weighing scale for demonstrating load cell measurements.

A simple weighing scale was constructed by 3D printing a stand for fixing the load cell on one end, and a weighing pan attached to the other end of the load cell, as shown in Fig. 14. The load cell has four leads attached. One pair of leads is for applying excitation voltage or current. The other pair of leads is for measuring voltage. The WE and CE connections from the potentiostat were connected to the excitation leads of the load cell. The RE and SE connections were attached to the voltage measurement leads. The MYSTAT was placed in galvanostatic mode and a fixed current of 10 mA was applied to the excitation leads of the load cell. The voltage drop between the legs of the Wheatstone bridge was recorded as known weights were added to the weighing pan. Total weight added to the pan ranged from 0 g to 200 g. It was found that the measured voltage increased linearly with added weight. The R-squared value of a linear fit of the measured voltage versus weight was found to be 0.9999. An unknown weight was placed on the pan and the linear calibration curve was used to calculate a weight from the measured potential. The weight determined from the load cell was found to be 68 g. A laboratory analytical balance determined the actual weight to be 67 g.

## 8 Conclusions

The MYSTAT potentiostat/galvanostat design was found to provide accurate, low noise measurements suitable for many standard DC electrochemical techniques used in research and development. The low cost and compact form factor also make the instrument attractive for teaching electrochemical techniques and how various electronic sensors work. The instrument offers higher current and voltage limits than previously published open source designs and comparable commercial instruments. The reported design enables facile assembly of a finished instrument in an enclosure without the need for custom machining. The main limitation of the device compared to commercial instruments is the relatively slow minimum measurement time of 90 ms, which is governed by the analog–digital converters chosen. Numerous options are available for improving the speed of the instrument by selecting different analog–digital converters in the design. The instrument is also limited to DC measurements. If the data collection rate were increased, it would also open up the possibility of adding AC measurement capability in future designs. There is currently a lack of general purpose AC potentiostats capable of electrical impedance spectroscopy in the open source hardware literature.

## Declaration of Competing Interest

The authors declare that they have no known competing financial interests or personal relationships that could have appeared to influence the work reported in this paper.
